# Primitive neuroectodermal tumor of the cervix diagnosed during pregnancy: a rare case report with discussion

**DOI:** 10.1186/s12884-021-03859-6

**Published:** 2021-05-18

**Authors:** Xiang Feng, Lina Zhang, Yanling Tan, Aihua Feng, Fei Luo, Mengfan Xu, Hong Ye, Hongyu Zhu, Peng Zhou, Hua Li

**Affiliations:** 1 Department of Gynaecology and Obstetrics, The First College of Clinical Medical Science, Three Gorges University, YiChang, 443000 China; 2 State Key Laboratory of Food Science and Technology, Jiangnan University, Wuxi, 214122 China; 3 Department of Orthopedics, The First Hospital of YiChang, YiChang, 443000 China

**Keywords:** Pregnancy, Cervix, Primitive neuroectodermal tumor, Treatment

## Abstract

**Background:**

Primitive neuroectodermal tumor (PNET) is a relatively rare malignant small round cell tumor, and the occurrence of cervical PNET during pregnancy is extremely rare.

**Case presentation:**

A case of pregnancy complicated by PNET at our hospital was reported. A 19-year-old pregnant woman presented to the hospital due to multiple instances of vaginal bleeding during the first and second trimesters. She was initially considered for threatened abortion but was ultimately diagnosed with cervical PNET. No standard treatment plan has been developed for pregnant women with this tumor. After completing the necessary examinations, doctors cooperated with the patient and her family to develop a surgical treatment plan. The patient recovered well after surgery, but she refused radiotherapy and chemotherapy. After nearly 3 years of follow-up visits, the patient is alive with no signs of recurrence.

**Conclusions:**

PNET during pregnancy is a rare but complex condition. It is necessary to devise an individualized treatment plan according to gestational age. Timely surgical treatment can significantly prolong the survival time of patients but may also lead to fetal loss and the inability to carry a pregnancy.

## Background

Primitive neuroectodermal tumor (PNET) is a highly malignant small round cell tumor that originates from the primitive neuroepithelium; this tumor type is primarily divided into central nervous system PNET (cPNET) and peripheral PNET (pPNET). Although PNET can arise in any part of the body, cervical PNET during pregnancy is rare. Here, a case of pregnancy complicated by primary cervical neuroectodermal tumor at our hospital is reported.

## Case presentation

The patient was 19 years of age and unmarried. She had her first sexual experience at the age of 18 and was gravida 1 and para 0 with no family history of tumors but a personal history of an ovarian tumor. At age 14, the patient underwent abdominal left ovarian tumor resection due to a borderline sex cord-stromal tumor of the left ovary. No other treatment was administered, and no obvious abnormalities were observed during regular follow-up visits. The patient’s last menstruation was February 15, 2017. She was diagnosed with early intrauterine pregnancy by uterine Doppler ultrasound on April 5, 2017, and the gestational age was approximately 7 weeks. The patient had no obvious history of irregular vaginal bleeding and no abdominal pain or distension before pregnancy. At approximately 9 weeks gestation, the patient experienced a small amount of vaginal bleeding but no abdominal pain. She visited the local hospital several times because of repeated vaginal bleeding. As she was considered to have threatened abortion, the patient was treated for threatened miscarriage.

At 14 weeks, the patient presented to the local hospital due to sudden bright red vaginal bleeding. Tocolytic therapy was administered with no significant effects. At 15 weeks, vaginal bleeding recurred with slight abdominal pain and distension. A gynecological examination was performed, and a small amount of odorless bloody fluid was observed within the vagina. The opening of the cervix was blocked by a 3 × 3 cm lobulated mass, which was gray-red in color with a thick base and poor mobility. The bleeding was reduced after the neoplasm was removed. After examination, the neoplasm was diagnosed as a cervical malignant tumor with special tumor cell properties. A pathological consultation with a higher center was conducted, and the following immunohistochemical markers were observed: CD56 (strong +), α-synuclein (Syn) (partly +), CD99 (+), and Ki-67 70% (+). The diagnosis of cervical PNET was confirmed and was consistent with a poorly differentiated neuroblastoma.

The patient was transferred to our hospital on June 23, 2017. Fetal Doppler ultrasound showed a single live fetus approximately 19+ weeks. Alpha fetoprotein (AFP), carcinoembryonic antigen (CEA), and squamous cell carcinoma antigen (SCCA) were within normal ranges, but cancer antigen 125 (CA125) was slightly increased (43.2 U/ml). Gynecological examination revealed vaginal patency, a small amount of white discharge with no peculiar smell, a soft cervix, visible erosion in the 5/6/7 o’clock direction, positive contact hemorrhages, and no obvious adhesion near the uterus. The corpus uteri was in the middle, and the size of the uterus corresponded to the gestational period. The fundus was approximately two fingers below the umbilicus, with activity and no significant tenderness in bilateral accessory areas. The following primary diagnosis was made at the hospital: (1) pregnancy with cervical malignant tumor I B1 (pPNET); (2) intrauterine pregnancy at 18 weeks + 2 days; and (3) history of a borderline ovarian tumor after surgery.

**Fig. 1:**
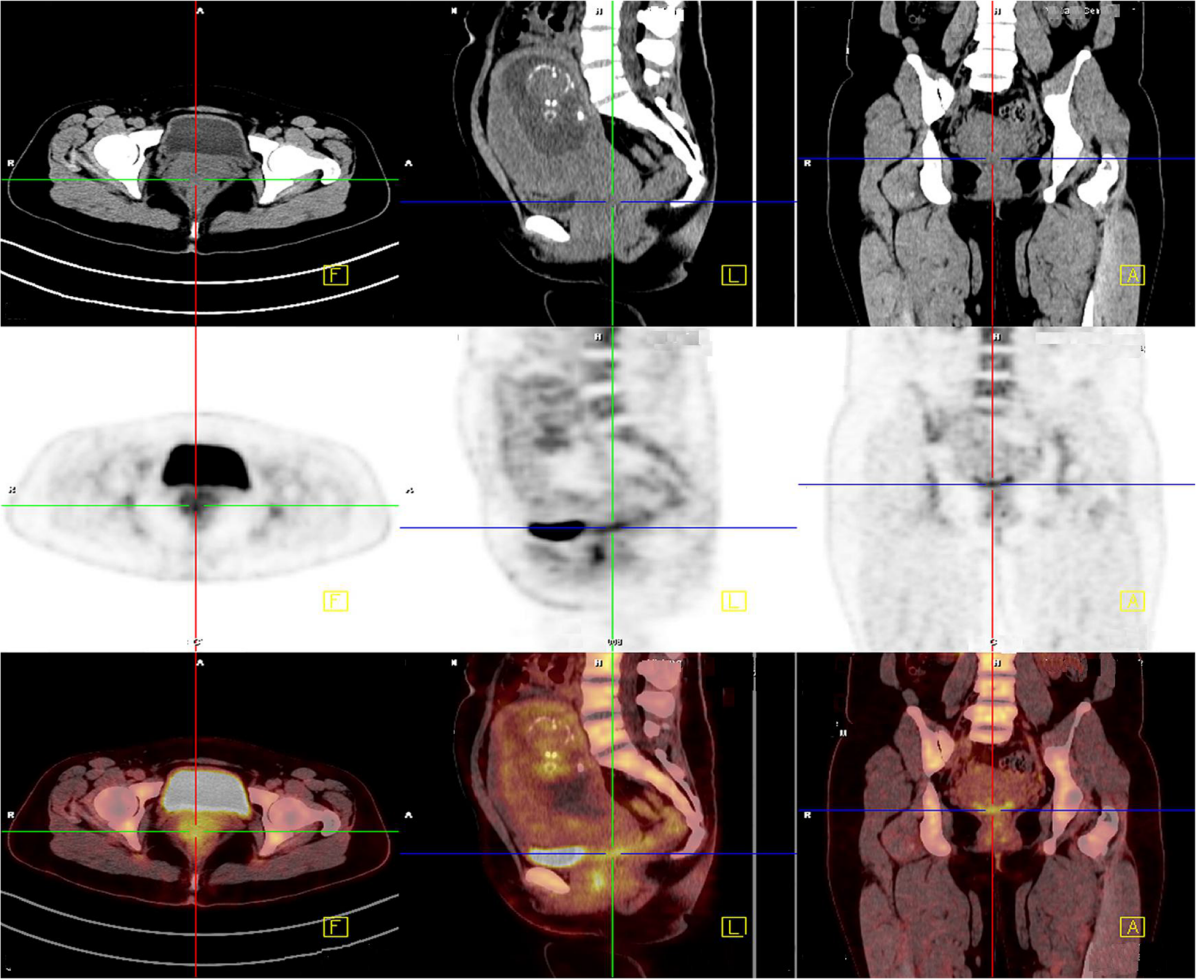
PET-CT examination revealed that the patient’s cervix was thickened and that her metabolism was increased. No obvious tumor was seen in other areas

**Fig. 2:**
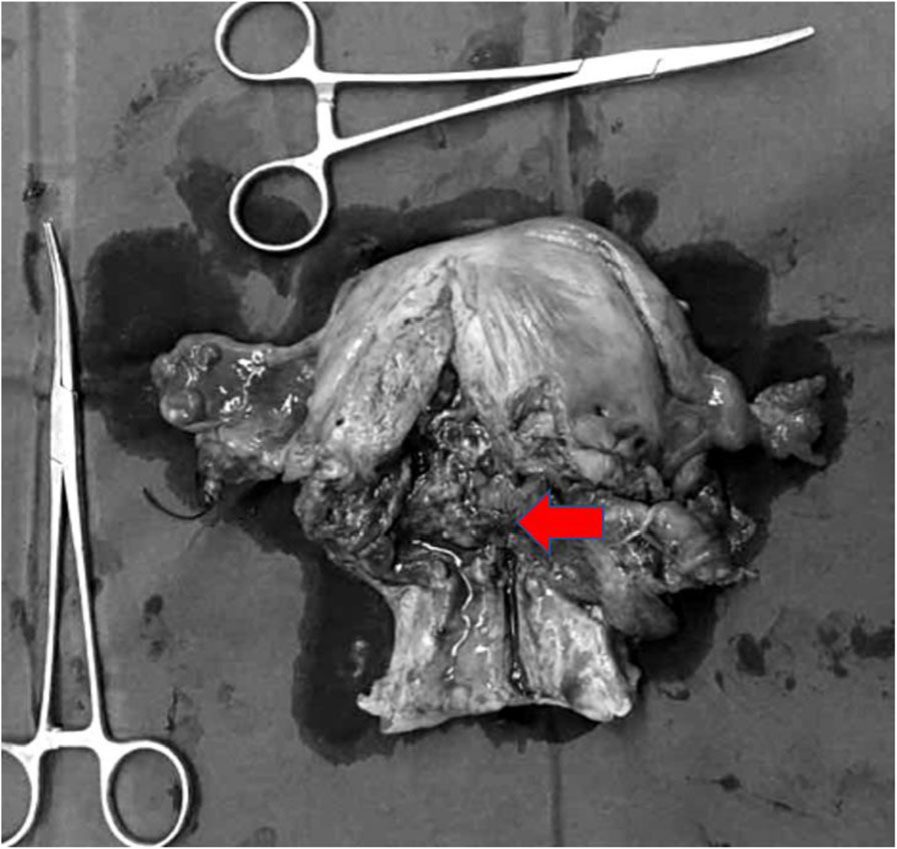
Macroscopic view of the resected uterus. The arrow indicates the location of the cervical tumor

Considering the special history and tumor particularity of the patient, we communicated several times with the patient and her family about the illness and feasible treatments, surgical options for preserving ovaries were also proposed. While the patient and her family insisted on radical hysterectomy to abandon the fetus and the simultaneous removal of the bilateral ovaries, they also understood that the patient would lose fertility and that the tumor could recur in other parts of the body. The PET-CT examination indicated an enlarged uterus and cervix as well as increased metabolism, but no abnormalities in other parts of the tissue were noted (Fig. 1). On July 3, 2017, modified radical hysterectomy with bilateral adnexectomy was performed, and during the surgery, the uterus was the appropriate size for the gestational month, the cervical canal was slightly thickened, and no obvious lymph node enlargement was observed (Fig**.** 2). The patient recovered well after surgery.

**Fig. 3:**
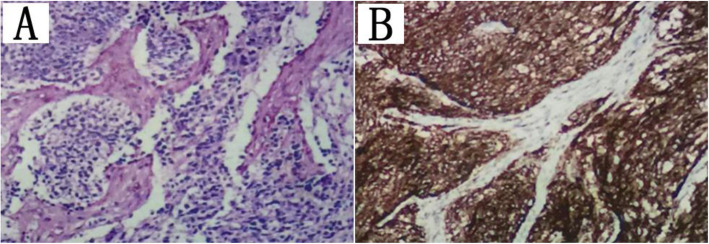
Section of the cervix showing (**a**) tumor cells arranged in the form of sheets (hematoxylin–eosin [HE] × 400). **b** Immunohistochemistry showing tumor cells positive for CD99 (× 400)

Immunohistochemistry revealed that the tumor was CD99 (+), Ki-67 (LI: 70%), CD34 (−), and vimentin (partly+) (Fig. 3). The pathological diagnosis was PNET with no lymph node metastasis. Because the patient had a history of multiple tumors, we suggested tumor genetic testing to determine the presence of genetic or chromosomal abnormalities (e.g., tumor suppressor genes, the susceptibility genes BRCA1 and BRCA2) and to determine whether they are associated with the morbidity of these two tumor types, but this recommendation was rejected. Outpatient follow-up visits lasted for approximately 3 years, and no abnormalities were found. Outpatient follow-up visits are ongoing.

## Discussion

PNET is a rare small round cell malignancy that belongs to the same family as Ewing’s sarcoma. Dehner (1986) first proposed the concepts of cPNET and pPNET [1]. cPNET primarily refers to a type of malignant PNET that originates from supratentorial brain tissue and the spinal cord. pPNETs originate from the residue of early cell components or a primitive matrix of small round cells in the extracranial soft tissue, skeletal system or primitive neural groove and are collectively referred to as pPNETs, which have similar morphological and histological characteristics to the Ewing’s sarcoma family of tumors [2, 3]. pPNETs often occur in the extracranial musculoskeletal system and rarely occur in parenchymal organs. They primarily affect children and adolescents, with no significant difference between sexes. pPNETs have a high degree of malignancy, and the imaging findings are not specific. Therefore, the diagnosis depends on histology, immunohistochemistry, gene testing and electron microscopy [4, 5].

PNET in the cervix is an extremely rare type of pPNET. Approximately 20 patients were described in the literature from 1987 to 2019, and of those, 10 had a history of pregnancy [6]. Our patient, who was only 19 years of age, was also a rare case of pregnancy complicated by cervical pPNET.

The main clinical manifestations of primary cervical PNET reported in the literature are vaginal bleeding, lower abdominal pain and cervical masses [6–8]. Gynecological examination showed that the cervix resembled a cauliflower or a nodular mass growing outward, which was difficult to distinguish from cervical squamous cell carcinoma [6–9]. The clinical manifestations of this case were vaginal bleeding and cervical masses. However, interference with the clinical diagnosis was increased because the patient was pregnant, which made the diagnosis difficult.

The microscopic features of pPNET are primarily small round cells with little cytoplasm and immature differentiation, but necrosis and nuclear fission are also commonly observed. Moreover, Homer-Wright chrysanthemums may be observed in pathological sections. Immunohistochemistry typically indicates CD99, vimentin, CD56 and Syn positivity. Most scholars believe that the Homer-Wright chrysanthemum structures and the presence of neuroendocrine particles containing at least two different neural markers seen by electron microscopy or immunohistochemistry are important evidence for the diagnosis of pPNET [10, 11]. PNET tissue of the uterus originates from (1) the migration of neural crest and neural tube cells in the embryo, (2) fetal neuroectodermal tissue, or the (3) Mullerian tube. It was temporarily impossible to determine the source of pPNET in our case, as all three sources were possible. Although the origin of cervical PNET is not clear, the immunohistochemical markers of cervical neoplasms in this case, including CD99, Syn, CD56, and vimentin (partial), were positively expressed, which satisfies the diagnostic criteria of pPNET. The expression of Ki-67 was as high as 70% in our case, which suggested active cell proliferation and a poor prognosis.

Standard treatment for pPNET of the cervix has not yet been established. However, the ideal treatment method is adjuvant chemotherapy after diagnosis, followed by complete surgical resection, postoperative adjuvant radiotherapy and chemotherapy. The surgical resection range is based on soft tissue tumor resection and extensive resection, which are performed in the same manner as in Ewing’s sarcoma and other soft tissue sarcomas; the resection includes the tumor and approximately 3–5 cm of surrounding normal tissue [12]. pPNET is sensitive to radiotherapy, which is an important postoperative adjuvant therapy that may reduce local recurrence [6]. Nevertheless, a standard treatment for PNETs of the cervix has not been established.

In this case, the tumor was accompanied by pregnancy, and the patient experienced repeated vaginal bleeding at the time of treatment. The amount of bleeding gradually increased after tocolytic therapy. Therefore, the present case provides obstetricians and gynecologists with the suggestion that vaginal bleeding symptoms are easily misdiagnosed as threatened abortion or premature delivery and should not be ignored during pregnancy. Timely gynecological examination during pregnancy is also crucial. Notably, our patient was pregnant at only 19 years of age, which introduced difficulties in the treatment, and she had a limited window for surgical treatment because the cervical pPNET was highly malignant. The surgical procedure required hysterotomy to remove the fetus, but the fetus was small and did not survive. Patients who undergo modified radical hysterectomy and bilateral adnexectomy permanently lose fertility. This choice is difficult from a medical and ethical perspective. During the treatment process, it is necessary to communicate repeatedly with patients and their families about treatment plans and related risks and to respect the wishes of the patients and their families during the formulation of individualized treatment plans.

Like other sarcomas, PNETs typically metastasize via the blood, but they may also metastasize via the lymphatic system. Approximately 30 to 50% of patients have distant metastases at the initial diagnosis, which results in a significant reduction in survival time [13]. Although surgical treatment for pPNET of the cervix primarily involves radical hysterectomy, lymph node dissection was also performed in this case because pPNET can metastasize through the lymph nodes. Postoperative pathology confirmed that this PNET did not metastasize via the pelvic lymph nodes, but this finding did not indicate that lymph node dissection was unnecessary in the surgical treatment of cervical pPNET in the protocol.

According to the characteristics of pPNET and the treatment plan, radiotherapy and chemotherapy should be administered after surgery. Unfortunately, the patient adamantly refused chemotherapy, which increased the possibility of pPNET recurrence after surgery. This patient is currently alive more than 3 years after surgery. No abnormal symptoms or positive signs were observed during follow-up. No abnormalities were found on imaging, but the follow-up observation time should be extended. The patient was satisfied with the entire treatment process.

## Conclusions

PNET is highly malignant and has a poor prognosis. Pregnancy combined with cervical PNET is very rare, and no standard treatment has been established. Therefore, it is necessary to continuously summarize multiple experiences to provide a reliable clinical basis and plan for the treatment of this disease. We formulated a feasible treatment plan in the present case. Whether a patient may have a better pregnancy outcome requires further investigation.

## Data Availability

All data generated or analyzed during this study are included in this article.

## Abbreviations

PNET: Primitive neuroectodermal tumor
cPNET: Central primitive neuroectodermal tumor
pPNET: Peripheral primitive neuroectodermal tumor
AFP: Alpha fetoprotein
CEA: Carcinoembryonic antigen
SCCA: Squamous cell carcinoma antigen
CA125: Cancer antigen 125

## References

[1] Dehner LP (1986). Peripheral and central primitive neuroectodermal tumors. A nosologic concept seeking a consensus. Arch Pathol Lab Med.

[2] Ganugapanta L, Pendem S, Chatni S, Patil BR (2015). Mandibular peripheral primitive neuroectodermal tumor: a rare case report with review of literature. J Maxillofac Oral Surg.

[3] Kampman WA, Kros JM, De Jong TH, Lequin MH (2006). Primitive neuroectodermal tumours (PNETs) located in the spinal canal; the relevance of classification as central or peripheral PNET : case report of a primary spinal PNET occurrence with a critical literature review. J Neuro-Oncol.

[4] Ke C, Duan Q, Yang H, Zhu F, Yan M, Xu SP (2017). Meningeal Ewing sarcoma/peripheral PNET: Clinicopathological, Immunohistochemical and FISH study of four cases. Neuropathology..

[5] Rekhi B, Vogel U, Basak R, Desai SB, Jambhekar NA (2014). Clinicopathological and molecular spectrum of Ewing sarcomas/PNETs, including validation of EWSR1 rearrangement by conventional and array FISH technique in certain cases. Pathol Oncol Res.

[6] Khosla D, Rai B, Patel FD, Sreedharanunni S, Dey P, Sharma SC (2014). Primitive neuroectodermal tumor of the uterine cervix diagnosed during pregnancy: a rare case with review of literature. J Obstet Gynaecol Res.

[7] Pauwels P, Ambros P, Hattinger C, Lammens M, Dal Cin P, Ribot J (2000). Peripheral primitive neuroectodermal tumour of the cervix. Virchows Arch.

[8] Khwaja R, Mantilla E, Fink K, Pan E (2019). Adult primary peripheral PNET/Ewing's sarcoma of the cervical and thoracic spine. Anticancer Res.

[9] Ahmad I, Chufal KS, Bhargava A, Bashir I. Primitive neuroectodermal tumour of the cervix: a rare diagnosis. BMJ Case Rep. 2017;2017:bcr2016217461. 10.1136/bcr-2016-217461, https://pubmed.ncbi.nlm.nih.gov/28052947/. 10.1136/bcr-2016-217461 PMC5256460 28052947

[10] Sen S, Kashyap S, Thanikachalam S, Betharia SM (2002). Primary primitive neuroectodermal tumor of the orbit. J Pediatr Ophthalmol Strabismus.

[11] Rothenberg AB, Berdon WE, D'Angio GJ, Yamashiro DJ, Cowles RA (2009). Neuroblastoma-remembering the three physicians who described it a century ago: James Homer Wright, William pepper, and Robert Hutchison. Pediatr Radiol.

[12] Subbiah V, Anderson P, Lazar AJ, Burdett E, Raymond K, Ludwig JA (2009). Ewing's sarcoma: standard and experimental treatment options. Curr Treat Options in Oncol.

[13] Windsor R, Strauss S, Seddon B, Whelan J (2009). Experimental therapies in Ewing's sarcoma. Expert Opin Investig Drugs.

